# The Metropolitan Hospital

**Published:** 1892-07-16

**Authors:** 


### THE METROPOLITAN HOSPITAL.

THE annual dinner in aid of the funds of the Metropolitan Hospital, Kingsland Road, Shoreditch, was held on the 30th ult., in the Whitehall Rooms of the Hotel Metropole, under the presidency of Lord Sandhurst. The company, who numbered about one hundred, included Mr. T. W. Wheeler, Q.C., Mr. Joseph Fry, Mr. Dyer Edwardes, Rear-Admiral Bowden-Smith, Lieutenant-Colonel E. Montefiore, Mr. R. T. Wragg, Mr. J. R. Pike, the Rev. R. H. Hadden, Mr. Coleman Defries, Mr. F. Snowden, Mr. H. H. Tooth, and the Secretary, Mr. C. H. Byers.

The CHAIRMAN, in proposing the toast of the evening, took occasion to dwell upon the services rendered to the hospital by Mr. Joseph Fry, a son of Mrs. Elizabeth Fry, who had extended so keen and practical a sympathy to the poor and the lowly. Such an institution as the Metropolitan Hospital, he added, had peculiar claims to support. It was in the midst of a very poor districc, comprising Bethnal Green, Haggerston, Shoreditch, and Dalston. Apart from the German Hospital, it was two miles away from any other charity of the kind. Secondly, the Committee adopted the provident system, thereby increasing the encouragement afforded to thrift. The hospital was at present in need of a greatly-enlarged support. It had 160 beds, and more than half of these were compulsorily vacant. Cynics said: "But if you gave, no one would pay"; but they spoke without sufficient knowledge. He had had the privilege of presiding over the House of Lords' Committee on the subject of Poor-law relief and charity, and was glad to have an opportunity of thanking the officials of metropolitan charities and the Charity Organisation Society for the evidence they had given when it was required. The Committee had just issued their report, which showed that, in their opinion, the management of hospitals was generally good. No serious complaints had been brought before the Committee. The hospitals of London had emerged from the enquiry with great honour, but he was strongly of opinion that some sort of central board was desirable, and that if the public could only be assured that the hospitals were administered properly, their funds would be tremendously augmented. The public at large might not be well acquainted with the subject, but, unless they subscribed more liberally to the funds of these institutions, the time might come when Government aid or municipal subventions would have to be applied for.

Mr. JOSEPH FRY, who met with a cordial greeting, replied to the toast.

Other toasts followed, and subscriptions to the amount of £2,472, which included £1,000 to endow a cot in memory of the late John Carter, were announced by the Secretary.

EVERYONE is aware that the diplomacy of the world is, and always has been, regulated by the fair sex; but they now have a serious rival in their way, at least in semi-civilised countries. The conjuror has been pressed into the service of diplomatists with excellent effect by the English Foreign Office in its dealings with Morocco. No African exploring expedition of the future will be complete without adding to its menage this resource of civilisation; the influence of a clever conjuror on the minds of the simple natives would be more efficacious, and far more consonant with humanity than powder and shot, to which they are too often treated.

